# Eye diseases associated with psoriatic arthritis in the Amazon

**DOI:** 10.5935/0004-2749.20220086

**Published:** 2025-08-22

**Authors:** Thaís Suellen Ramos Allen, Jaqueline Azevedo Leão, Bruna Cavaleiro de Macêdo Souza, Ana Carolina Batista Pamplona de Freitas, Robson Seiji Tsuchiyama Koyama, Roberta Vilela Lopes Koyama

**Affiliations:** 1 Universidade Estadual do Pará, Belem, PA, Brazil; 2 Hospital Nipo Brasileiro de Olhos, Belem, PA, Brazil

**Keywords:** Psoriatic arthritis, Eye manifestation, Keratoconjunctivitis sicca, Dry eye syndrome, Blepharitis, Artrite psoriásica, Manifestação ocular, Ceratoconjuntivite seca, Síndrome do olho seco, Blefarite

## Abstract

**Purpose:**

Ocular disorders are among the most frequent manifestations of psoriatic arthritis. The incidence, type, and severity of these disorders may be influenced by genetics, local environmental factors, and access to ophthalmic treatment. Here we describe the ocular manifestations of psoriatic arthritis among denizens of the Amazon region of Para, Brazil, treated by the rheumatology service of *Universidade do Estado do Pará*.

**Methods:**

This cross-sectional study examined 23 psoriatic arthritis patients (median age 47.78 years, no sex predominance) diagnosed according to Caspar’s criteria. Disease activity was evaluated according to the Clinical Disease Activity Index for Psoriatic Arthritis. Ophthalmological examinations performed included visual acuity with distance correction, biomicroscopy, applanation tonometry, fundoscopy, Schirmer test I, tear breakup time, fluorescein staining, and lissamine green staining. Patients also completed The Ocular Surface Disease Index questionnaire.

**Results:**

The most common ophthalmic disorders were dry eye (60.9%), cataracts (56.5%), blepharitis (47.8%), keratitis (43.5%), meibomitis (30.4%), pterygium (26, 1%), and pinguecula (13%). More than half of all patients demonstrated recent onset (>5 years), the peripheral disease type, and severe symptoms according to Clinical Disease Activity Index for Psoriatic Arthritis.

**Conclusion:**

The ocular manifestations of psoriatic arthritis are varied and mainly affect the ocular surface. Regular ophthalmological follow-up is recommended for patients in the early stage with high disease activity.

## Abstract (pt)

**Objetivo:**

Descrever as manifestações oftalmológicas observadas em pacientes com artrite psoriásica atendidos no Serviço de Reumatologia da Universidade do Estado do Pará.

**Métodos:**

Estudo transversal. A amostra foi composta por 23 pacientes com artrite psoriásica, segundo os critérios de Caspar, atendidos no ambulatório de reumatologia da Universidade do Estado do Pará. Para avaliação da atividade de doença foi aplicado o *Clinical Disease Activity index for Psoriatic Arthritis* e, posteriormente, foi realizado o exame oftalmológico (acuidade visual com correção para distância, biomicroscopia, tonometria de aplanação de Goldmann, fundoscopia, teste de Schirmer I, tempo de ruptura do filme lacrimal, lissamina verde e questionário The Ocular Surface Disease Index).

**Resultados:**

Nesta pesquisa não houve predomínio entre os sexos e a população tinha uma mediana de idade de 47,78 anos. As manifestações mais comuns foram olho seco (60,9%), catarata (56,5%), blefarite (47,8%), ceratite (43,5%), meibomite (30,4%) e pterígio (26,1%).

**Conclusão:**

As manifestações oculares encontradas em pacientes com artrite psoriásica são variadas e afetam sobretudo a superfície ocular; no entanto, são clinicamente subestimadas; por isso recomenda-se o acompanhamento oftalmológico regular e periódico para pacientes com artrite psoriásica.

## INTRODUCTION

Psoriatic arthritis (PsA) is a chronic inflammatory arthropathy associated with psoriasis^(1)^ in 4%-30% of patients^(2)^. In Brazil, PsA is the second most frequent spondyloarthropathy, accounting for 13.7% of total cases^(1)^. Among the most important extra-articular manifestations are ocular symptoms, which are observed in about 31% of PsA patients^(3)^ and may be directly associated with the disease or secondary to treatment^(4)^. The most frequently observed ophthalmic complications in previous study cohorts were conjunctivitis (19.6%), uveitis (5%), blepharitis (12.5%), cataracts (10%), glaucoma (10%), superficial punctate keratitis (22.5%), pinguecula (20%), and keratoconjunctivitis sicca (15%)^(5,6)^.

However, relatively few studies have evaluated the prevalence and characteristics of specific eye diseases in PsA patients^(5)^. As ophthalmic disorder distribution in PsA may differ according to ethnicity, geographic region, and access to treatment, this study examined ocular manifestations among PsA patients treated at a medical university in the Amazon region of Brazil.

## METHODS

### Study design and approval

This cross-sectional study was approved by the Research Ethics Committee of the Universidade do Estado do Pará (number: 3.311.461). The minimum sample size calculation was performed using OpenEpi, version 3.01 (htrps://www.openepi.com). According to the calculation, a minimum sample size of 21 was deemed necessary to detect an ocular manifestation prevalence of 30% among PsA patients with a precision margin of 20% based on an infinite patient population, a design effect equal to one, and confidence interval (CI) of 95%. This study included 23 patients with PsA receiving treatment at the rheumatology clinic of UEPA (Belém, Para, Brazil) from March 2019 to January 2020. Inclusion criteria were 18 years of age or older, PsA diagnosis confirmed by a ClASsification criteria for Psoriatic ARthritis (CASPAR) score ≥3, and providing informed written consent. Patients with sarcoidosis, Sjogren’s syndrome, systemic lupus erythematosus, Behçet’s disease, rheumatoid arthritis, neoplasia, inflammatory bowel disease, infectious diseases, eye trauma, eye allergy, abnormal movement of eyelids, pathologies susceptible to ocular involvement, and previous eye surgery were excluded from the study.

### Examinations

An initial clinical interview was conducted using a specific questionnaire created by the authors evaluating clinical and demographic characteristics. Disease activitye was evaluated using the Clinical Disease Activity Index for Psoriatic Arthritis (cDAPSA), which classifies patients according to total score as follows^(7)^: remission (≤4), low disease activity (>4 and ≤13), minimum disease activity (>13 and ≤27), and high disease activity (>27). Subsequently, patients were referred to Hospital Nipo de Olhos (HNOlhos), an ophthalmological hospital in Belém, where the following examinations were conducted by the same ophthalmologist: visual acuity with distance correction, biomicroscopy, Goldman tonometry, fundoscopy, Schirmer test I, tear film break-up time (TBUT), fluorescein staining, and lissamine green staining. Patients also completed the Ocular Surface Disease Index (OSDI) questionnaire.

Visual acuity (VA) was measured using the decimal denotation table at a distance of six meters and classified as normal if VA correction ≥0.8 in one or both eyes for all ages or deficient if ≤0.7 in one or both eyes^(8)^. A Goldman applanation tonometer was used to assess intraocular pressure (IOP), with values from 11 to 21 mmHg considered normal^(9)^. Biomicroscopy exams were performed to assess changes in the eyelids, eyelashes, conjunctiva, cornea, anterior chamber, iris, lens, and anterior vitreous. Fundoscopy was performed with the aid of a 90-diopter Volk lens to assess the macula, vessels, and optic nerve.

Tear film stability was evaluated by tear breakup time (TBUT) using a drop of 1% fluorescein. After application of fluorescein to the corneal surface, the patient was instructed to blink several times to equalize the dye distribution. The corneal surface was then examined by lit lamp with the cobalt blue filter to identify the appearance of the first dry spot after the last blink. The time to appearance was measured using a digital stopwatch. The average of three separate trials was recorded, and a mean greater than 10 seconds was regarded as normal. The corneal surface was also evaluated right after the TBUT by slit lamp for the presence or absence of punctate keratopathy. Dry eye was further examined by placing a lissamine green strip in contact with the lacrimal meniscus of the bottom sac for 1 minute. The reaction was classified according to the Van Bijsterveld Scoring System ^(10)^. The Schirmer test I was conducted without topical anesthetic using a standardized Whatman no.41 filter paper strip (35 mm long by 5 mm wide). A tear travel distance less than or equal to 10 mm were considering abnormal^(11)^. Symptoms of dry eye were further assessed according to OSDI questionnaire scores as follows^(12)^: normal (0-12), mild dye eye (13-22), moderate dry eye (23-32), severe dry eye (33-100). Dry eye was diagnosed according to *The Japanese criteria for dry eyes*^(13)^ (2016), where OSDI score ≥13 and average TBUT ≤5 s is considered positive ^(12,13)^.

All graphs and tables were constructed using Microsoft Word, Excel, or BioEstat 5.5. Statistical analysis was performed using BioEstat 5.5 with the aid of a biostatistician. The associations between categorical variables were assessed using the G test. Standardized residue analysis was performed to identify those frequencies that contributed most to significant results. The Mann-Whitney U test was used to compare quantitative variables between patient subgroups. A p≤0.05 (two-tailed) was considered statistically significant for all tests.

## RESULTS

Twenty-three patients participated in the study, 16 females (69.6%) and 7 males (30.4%). Of these 23 patients, 52.2% were X. The mean age of the study population was 57.7 years (SD 14.8), and there was not significant differ between females (58.3 years, SD 13.2) and males (56.6 years, SD 19.1). The most frequent comorbidities were hypertension (47.8%), obesity (39.1%), and diabetes (26.1%). Fifteen patients (65.2%) reported psoriasis before arthritis, 4 (17.4%) reported arthritis before skin lesions, and 4 (17.4%) reported simultaneous onset. Other clinical characteristics are summarized in table 1.

**Table 1:** Clinical characteristics of psoriatic arthritis (PsA) patients examined by the Universidade do Estado do Pará rheumatology clinic

Clinical characteristics		Frequency	%	
PsA duration <5 years		12†	52.2	
5-20 years		8	34.7	
>20 years		3	13	
	Predominant Clinical Form			
	Peripheral	15^†^	65.2	
	Axial	4	17.4	
	Enthesitis	2	8.7	
	Dactylite	2	8.7	
cDAPSA Low disease activity		4	17.4	
Minimum disease activity		6	26.1	
High disease activity		13	56.5	

The G test was used for all comparisons.

† = Observed frequency higher than expected.

cDAPSA= Clinical Disease Activity Index for Psoriatic Arthritis. **Source:** Research questionnaire.

Of the 23 patients, 13 (56.5%) were receiving methotrexate treatment alone, one (4.3%) an immunobiological alone, 6 (26.1%) both methotrexate and an immunobiological, and three (13.0%) were not receiving any drug treatment at the time of the survey. There was no statistically significant association between drug regimen and ocular manifestations.

Twenty-two of 23 patients (95.7%) reported at least one of the three primary ophthalmic conditions, biomicroscopy abnormalities, dry eye, and keratitis. Ophthalmological findings are described in table 2. Twenty-one patients (91.3%) demonstrated low TBUT (<10 s), 19 (82.6%) an OSDI score ≥13, 11 (47.8%) an abnormal Schirmer I test (≤10 mm), 10 patients (43.5%) abnormal fluorescein staining, and 4 patients (17.4%) abnormal lissamine green staining. There were no statistically significant associations between OSDI scores and eye test results or cDAPSA clinical activity classification (Table 3).

**Table 2:** Most frequent ocular manifestations among PsA patients

Variable	Frequency	%
Meibomitis	7	30.4
Blepharitis	11	47.8
Pterygium	6	26.1
Cataract	13	56.5
Dry eye (2016 criteria)	14	60.9
Unilateral	8	34.8
Bilateral	6	26.1
Keratitis	10	43.5

**Source:** Research questionnaire.

**Table 3:** Associations between Ocular Surface Disease Index (OSDI) questionnaire classification and cDAPSA classification

Variable	General	Low activity (n=4)	Minimum activity (n=6)	High activity (n=13)	p-value
OSDI					0.022
Normal	4 (17.4)	3 (75.0)^†^	0 (0.0)	1 (25.0)	
Mild	1 (4.3)	0 (0.0)	1 (100.0)	0 (0.0)	
Moderado	6 (26.1)	1 (16.7)	1 (16.7)	4 (66.7)	
Severe	12 (52.2)	0 (0.0)^*^	4 (33.3)	8 (66.7)	

Categorical variables are represented as n (%) relative to the column total.

The G test was used for all comparisons.

* = Observed frequency lower than expected.

† = Observed frequency higher than expected. **Source:** Research questionnaire.

Among the 7 patients who presented with meibomitis, 4 (57.1%) had severe symptoms according to the OSDI questionnaire, 2 (28, 6%) moderate symptoms, and 1 (14.3%) normal or mild symptoms. Among the 11 patients with blepharitis, 5 (45.5%) reported severe symptoms, 4 (36.4%) moderate symptoms, and 2 (18.2%) normal or mild symptoms.

Of the 23 patients with PsA evaluated in the study, 14 (60.8%) had dry eye, and there were no correlations with other clinical characteristics (Table 4). There were also no statistically significant associations between the frequencies of specific ocular findings and cDAPSA classification, disease duration, presence of diabetes, and clinical form of PsA (Table 5).

**Table 4:** Associations of dry eye with biomicroscopy findings and keratitis

Variable	No dry eye (n=9)	Dry eye (n=14)	p-value
Biomicroscopy findings			
Meibomitis	2 (28.6)	5(71.4)	0.824
Blepharitis	4 (36.4)	7 (63.6)	0.867
Pterygium	3 (50.0)	3 (50.0)	0.883
Cataract	7 (53.8)	6 (46.2)	0.218
Keratitis	5 (50.0)	5 (50.0)	0.613

Categorical variables are represented as n (%) relative to the column total.

The G test was used for all comparisons. **Source:** Research questionnaire.

**Table 5:** Associations between the most frequent ocular manifestations and other clinical characteristics of psoriatic arthritis

Variable			Ocular manifestations			
		Cataract	Meibomitis Blepharitis		Keratitis	
Diabetes No		7 (53.8)^*^	6 (85.7) 6 (54.5)		7 (70.0)	
Yes		6 (46.2)†	1 (14.3) 5 (45.5)		3 (30.0)	
p-value		0.030	0.733 0.116		0.917	
	PsA duration					
	<5 years	6 (46.2)	3 (42.9)	5 (45.5)	4 (40.0)	
	5-20 years	5 (38.5)	2 (28.6)^†^	3 (36.1)	4 (40.0)	
	>20 years	2 (15.4)	2 (28.6)	2 (18.2)	2 (20.0)	
	p-value	0.820	0.027	0.696	0.576	
Predominant clinical form Axial		2 (16.7)	2 (28.6)	2 (18.2)	2 (20.0)	
Dactylite		1 (8.3)	0 (0.0)	1 (9.1)	0 (0.0)	
Enthesitis		0 (0.0)	1 (14.3)	1 (9.1)	1 (10.0)	
Peripheral		9 (75.0)	4 (57.1)	7 (63.6)	7 (70.0)	
p-value		0.343	0.466	0.999	0.485	

Categorical variables are represented as n (%) relative to the column total.

Blepharitis was defined as a non-zero score.

The G test was used for all comparisons.

* = Observed frequency lower than expected.

† = Observed frequency was higher than expected.

**Source:** Research questionnaire.

## DISCUSSION

Psoriatic arthritis is a heterogeneous disease with complex presentation, hampering the identification of specific risk factors. While distribution shows no sex predominance^(2)^ as observed in the present study, one study found that the disease is more common in Caucasians^(14)^. Here we found no statistical difference between Caucasian and X patients, but miscegenation is high among the Amazon population.

The main comorbidities of PA are metabolic syndromes (MSs), with systemic arterial hypertension being the most prevalent (47.8%), followed by obesity (39.1%) and diabetes mellitus (26.1%)^(15)^. Recent evidence suggests that PsA contributes directly to MS incidence as well as to other cardiovascular risk factors that increase atherosclerosis incidence with age^(15)^ as well as disease severity^(2)^.

Psoriatic arthritis has a heterogeneous clinical spectrum and patients can present overlapping clinical patterns such as peripheral arthritis (oligoarticular or polyarticular with or without distal interphalangeal involvement), enthesitis, dactylitis, and/or predominantly axial involvement^(16)^. Ritchlin and colleagues found that peripheral arthritis was the most common clinical manifestation^(17)^, in accord with the current study (p<0.001), but patients may exhibit more than one pattern simultaneously or a changing pattern with age and disease progression. A previous study of Brazilian patients found that the most common ocular manifestations were uveitis, conjunctivitis, blepharitis, and dry eye^(5)^, while a study of 112 patients by Lambert and Wright (1976)^(6)^ reported eye inflammation (31.2%), conjunctivitis (19.6%), iritis (7.1%), dry eye (2.7%), and episcleritis (1.8%). In this study, however, neither uveitis nor conjunctivitis was identified in any patient. This may be explained by the predominance of patients in the early stage of the disease (<5 years) or the by effects of drug treatment, as the vast majority were receiving a broad-spectrum immunosuppressive agent, a biologic, or both, which could have prevented more severe extra-ar ticular manifestations.

The most frequent ocular findings found in the present study were dry eye (60.9%), cataracts (56.5%), blepharitis (47.8%), keratitis (43.5%), meibomitis (30.4%), pterygium (26.1%), and pinguecula (13%), while other ocular manifestations were observed in only one or two patients. Although cataracts are often one of the most frequent ocular symptoms, lens abnormalities are generally considered an incidental effect of advanced age^(18)^. Chandran and colleagues^(19)^ found that 63% of PsA patients had bilateral cataracts, although there was no control group. Furthermore, diabetic patients also show a higher incidence of cataracts due to osmotic changes from acute glycemic decompensation, which induce refractive and accommodative changes in the lenses^(20)^. Thus, the high incidence of cataracts may have been an indirect consequence of diabetes.

Blepharitis was the third most common ocular manifestation (47%), consistent with previous studies^(21)^, as psoriasis can affect many areas of skin, including the eyelids^(18,21)^. Patients with blepharitis also presented with other ocular symptoms, possible because the accumulation of scales on the eyelid can cause considerable discomfort and symptoms such as burning and itching^(18)^. Similarly, dysfunction of the meibomian gland associated with posterior blepharitis is observed frequently in patients with psoriasis^(18)^, and could contribute to the symptoms of dry eye. Dysfunction of the accessory tear glands responsible for the tear film lipid component is associated with a higher evaporation rate, causing tear film instability and a cycle of hyperosmolarity and inflammation of the tear functional unit^(22)^. Several studies have already suggested that patients with severe obstructive dysfunction of the meibomian gland have tear film instability and consequent evaporative dry eye^(5,23)^, consistent with the findings of the present study. In previous diagnostic schemes, the vital color of the cornea or conjunctiva was considered critical for the diagnosis of dry eye; however, according to new criteria^(12)^, damage to the ocular surface is no longer necessary for a definitive diagnosis. Rather, only a combination of symptoms yielding OSDI ≥ 13 plus an unstable tear film (TBUT < 5 s) it is sufficient^(24)^. In this study, a substantial proportion of patients with dry eye also demonstrated meibomian dysfunction. However, dry eye is a multifactorial chronic disease with multiple risk factors such as inflammation, aging, increased osmolarity, and wind among others^(13)^.

The tests used to evaluate evaporative dry eye included the TBUT, which was used to analyze tear film stability. Tear film stability depends on epithelial integrity and the quality of all film layers^(13,24)^. Consistent with current findings, previous studies have reported significantly lower mean TBUT in patients with psoriasis and PsA^(25)^. Further, about half of the patients in this cohort demonstrated reduced tear production on the Schirmer I test. Although it is not mandatory, these data are important for the diagnosis of dry eye type with water deficiency and evaluation of eye damage, which can lead to epithelial defects and infection^(13)^.

Superficial punctate keratitis, primarily in the lower third of the corneal epithelium, was the most common corneal abnormality as evidenced by fluorescein staining. While different staining tests have yielded inconsistent results for the presence of keratitis, several studies have concluded that fluorescein is the preferred dye for bulbar conjunctival staining^(26)^. In addition, we used lissamine green as fluorescein and lissamine green may stain different cells and areas due to the difference in molecular weight^(27)^. Fluorescein appears to dye cells and areas that are less affected, while lissamine green appears to dye the most compromised areas^(28)^, so the combination may facilitation detection of keratitis from mild to severe PsA cases. Moreover, staining was conducted by a single experienced ophthalmologist to reduce variability.

Ophthalmological symptoms vary according to ocular pathology. Dry eye may cause a ‘gritty’ or ‘sandy’ sensation, burning, itching, visual turbidity, and excessive tearing^(29)^, manifestations found in most of the patients in this study. Dry eye is a multifactorial disease of the ocular surface characterized by a loss of lacrimal film homeostasis and accompanied by ocular symptoms caused by instability and hyperosmolarity of the lacrimal film, inflammation of the ocular surface, and sensorineural abnormalities^(29)^. Shimazaki (2018)^(12)^ found a significant relationship between dry eye symptoms and clinical signs. In the present study as well, symptoms as assessed by the OSDI questionnaire^(30)^ were correlated with ophthalmological signs of dry eye.

Ocular manifestations were more frequent among patients with high general disease activity according to cDAPSA criteria, although this relationship did not reach statistical significance, probably due to the small sample size. However, previous studies have documented increased extra-articular manifestations, including ophthalmological manifestations, in more severe PsA cases^(3)^. Unexpectedly, ocular manifestations were more frequent in patients with a recent diagnosis (the last 5 years), in contrast to the findings of Yan and colleagues^(31)^, perhaps because patients with recent diagnosis may have yet to receive optimal clinical treatment^(18)^. This result suggests that ophthalmic manifestations may be related to severity of inflammation. It is thus essential to monitor these patients in the early clinical phase for ophthalmic symptoms as these may be exacerbated over time.

Some patients also exhibited fundoscopy signs suggestive of glaucoma such as increased excavation and pallor of the optic nerve. Patients showing abnormally high IOP were referred for follow-up at the tertiary level for specialized investigation and management.

The ocular manifestations of PsA are varied and can afflict multiple parts of the eye. Further, the prevalence and severity of ophthalmic disorders in PsA may be underestimated, especially in early-stage patients with high disease activity.
